# Efficacy and safety of tocilizumab and baricitinib among patients hospitalized for COVID-19: a systematic review and meta-analysis

**DOI:** 10.3389/fphar.2023.1293331

**Published:** 2023-11-22

**Authors:** Jin Zhang, Xiongxiong Fan, Xiaoyu Zhang, Fengli Jiang, Yiping Wu, Beibei Yang, Xinghuan Li, Dong Liu

**Affiliations:** ^1^ Clinical Pharmacy Office, Baoji Central Hospital, Baoji, Shaanxi, China; ^2^ Department of Pharmacy, Xi’an Jiaotong University Health Science Center, Xi’an, Shaanxi, China

**Keywords:** COVID-19, immunomodulators, interleukin-6 inhibitors, JAK-STAT inhibitors, tocilizumab, baricitinib, efficacy and safety

## Abstract

**Introduction:** Tocilizumab and baricitinib are recommended treatment options for COVID-19 patients with hyperinflammatory response; however, there is a lack of systematic review directly evaluating their efficacy and safety.

**Objective:** This review was conducted to evaluate the efficacy and safety of tocilizumab and baricitinib in the treatment of hospitalized patients with COVID-19.

**Methods:** Relevant databases were searched for studies that compared the effect or safety of baricitinib or tocilizumab in hospitalized patients with COVID-19. The mortality was the main outcome. The hospital length of stay or adverse drug reactions were taken into consideration as secondary endpoints. The analyses were performed in Revman 5.3 or Stata 16.0. The protocol and analysis plan were pre-registered in PROSPERO, with the registration number CRD42023408219.

**Results:** In total, 10 studies with 2,517 patients were included. The overall pooled data demonstrated that, there was no statistically significant difference in the 28-day mortality rate and the hospital length of stay between the tocilizumab and baricitinib (OR = 1.10, 95% CI = 0.80–1.51, *p* = 0.57; OR = −0.68, 95% CI = −2.24–0.87, *p* = 0.39). The adverse reactions including secondary infection rate, thrombotic and bleeding events, and acute liver injury of tocilizumab were significantly higher than that of baricitinib. (OR = 1.49, 95% CI = 1.18–1.88, *p* < 0.001,OR = 1.52, 95% CI = 1.11–2.08, *p* = 0.009; OR = 1.52, 95% CI = 1.11–2.08, *p* = 0.009; OR = 2.24, 95% CI = 1.49–3.35, *p* < 0.001).

**Conclusion:** In patients hospitalized with COVID-19, no discernible difference in therapeutic efficacy was observed between tocilizumab and baricitinib; however, the group treated with baricitinib demonstrated a significantly lower incidence of adverse effects.

## Introduction

As the coronavirus disease 2019 (COVID-19) pandemic has spread across the globe, which has cost millions of lives (Zhu et al., 2020). Severe COVID-19 is always associated with a hyperinflammatory response, which results in the progressive release of proinflammatory cytokines, especially interleukin IL-6, IL-1, and IL-10, interferon, and tumor necrosis factor, provoking a “cytokine storm”. This response can further progress into acute respiratory distress syndrome, multiple organ failure, and even death (Mehta et al., 2020; Rabaan et al., 2021; Jain et al., 2023). The lack of clinically verified universal criteria makes it is difficult to treat the cytokine storm associated with COVID-19. Hence, in addition to antiviral medications (such as nirmatrelvir/ritonavir and remdesivir), immunosuppressive agents (including corticosteroids, tocilizumab, baricitinib, and anakinra) also play a crucial role in the therapeutic management of COVID-19 infection (Peng et al., 2022; Mengato et al., 2023a; Mengato et al., 2023b; Dahms et al., 2023). Corticosteroids are potent cytokine inhibitors, which gained evidence in reducing mortality and disease progression among COVID-19 patients on supplemental oxygen or mechanical ventilation (RECOVERY Collaborative Group et al., 2021). However, despite the administration of corticosteroids in some individuals, the cytokine storm persisted (Fajgenbaum and June 2020; Tang et al., 2021).

One of the major cytokines regulating the inflammatory response in COVID-19, serum IL-6 has been observed to correlate with mortality and has been proposed as a biomarker predictive of disease severity (Liu et al., 2020; McElvaney et al., 2021). Tocilizumab is a recombinant humanized monoclonal antibody against the human IL-6 receptor. Tocilizumab can effectively block cytokine storms by blocking the IL-6 signaling pathway, thereby inhibiting the human immune system and preventing immune cells from attacking human organs and causing damage (Stone et al., 2020; Rosas et al., 2021). Another therapeutic approach for managing COVID-19 is to target the Janus kinase/signal transducer and activator of the transcription (JAK/STAT) pathway, which mediates the signaling of multiple pro-inflammatory cytokines. Baricitinib is a JAK 1/2 inhibitor. Baricitinib interferes with the entry of viruses into host cells and can also block the JAK-STAT pathway to interfere with the antiviral response (Kalil et al., 2021; Marconi et al., 2021).

Numerous studies have demonstrated a lower risk of mortality and mechanical ventilation in severe COVID-19 patients with cytokine storms compared to standard of care (Gupta et al., 2021; Salama et al., 2021; Ely et al., 2022). According to current guidelines, tocilizumab and baricitinib should both be used in severe COVID-19 patients who have signs of systemic inflammation, preferably in conjunction with concurrent corticosteroids (Infectious Diseases Society of America, 2022; COVID-19 Treatment Guidelines Panel, 2019; World Health Organization, 2021). Therefore, which was superior between tocilizumab and baricitinib is still unclear. Then We conducted a meta-analysis to evaluate the efficacy and safety of tocilizumab and baricitinib in treating severe COVID-19 patients.

## Methods

This systematic review was performed according to the preferred items for systematic reviews and meta-analyses 2020 guideline (Page et al., 2021). The protocol and analysis plan were preregistered in PROSPERO, with the registration number CRD42023408219 (http://www. crd. york.ac.uk/PROSPERO/). In our report, no ethical approval was needed as this article is based on previously published article.

### Study design

The inclusion criteria encompassed randomized controlled trials or cohort studies that assessed the efficacy and safety of baricitinib *versus* tocilizumab in hospitalized patients with a clinical diagnosis of COVID-19. Trials that incorporated the combination of baricitinib with tocilizumab or had a follow-up duration shorter than 28 days were excluded from the analysis. Only articles with full-text access were considered, while conference proceedings, review articles, commentaries, etc., were excluded.

The primary outcome of this study was the comparison of the in-hospital mortality. The secondary outcomes were the duration of hospitalization, the duration of ICU stay, and the proportion of patients experiencing serious adverse events (SAEs) (including secondary infection rate, venous thromboembolic events, serious bleeding episodes, and acute liver injury).

### Search strategy

We conducted a comprehensive search for relevant studies up to April 2023 in PubMed, Embase, Cochrane Library, and the ClinicalTrials.gov website. Our search utilized keywords such as “COVID-19,” “SARS-CoV-2,” “Tocilizumab,” “Baricitinib,” “IL-6 inhibitors,” and “Janus kinase inhibitor.” The detailed search strategy can be found in Supplementary Appendix S1. Additionally, we identified additional references by thoroughly examining the reference lists of included studies and relevant reviews. Two independent reviewers were involved in the selection process based on title and abstract screening. Any disagreements were resolved through discussion with a third reviewer. Titles and abstracts were carefully screened, with full-text review undertaken when necessary due to ambiguity after reading the abstracts.

### Data extraction and quality assessment

Using a consistent data extraction spreadsheet, two writers separately extracted the data. The basic features of the articles, population, intervention, comparison group, and outcome of interest were all included in the retrieved data for pooling. No assumptions or oversimplifications were used when extracting the data.

The methodological qualities of all included RCTs were assessed using Cochrane’s tool for bias assessment, which encompassed six specific domains including selection bias, performance bias, detection bias, attrition bias, reporting bias, and other sources of bias. Each domain was classified as low risk of bias, high risk of bias, or unclear risk of bias (Higgins and Green, 2011). The methodological quality of included cohort studies was evaluated using the Methodological Index for Non-Randomized Studies (MINORS) (Slim et al., 2003), which consisted of 12 items with a maximum score of 24.

### Statistical analysis

The statistical analyses were conducted using Review Manager 5.3 software and Stata 16.0. The odds ratio (OR) and corresponding 95% confidence intervals (CIs) were utilized as outcome measures. Statistical significance was determined by a *p*-value less than 0.05, indicating the presence of statistically significant results. Heterogeneity among the studies was estimated using the I^2^ statistic, with pooled ORs calculated through either a fixed-effect model (in cases where heterogeneity was absent, I^2^ < 50%) or a random-effect model (when heterogeneity was present, I^2^ > 50%). Sensitivity analysis was performed employing the one-by-one exclusion method to assess the robustness of findings. Publication bias was evaluated utilizing an Egger funnel plot.

## Results

### Literature searching

**FIGURE 1:**
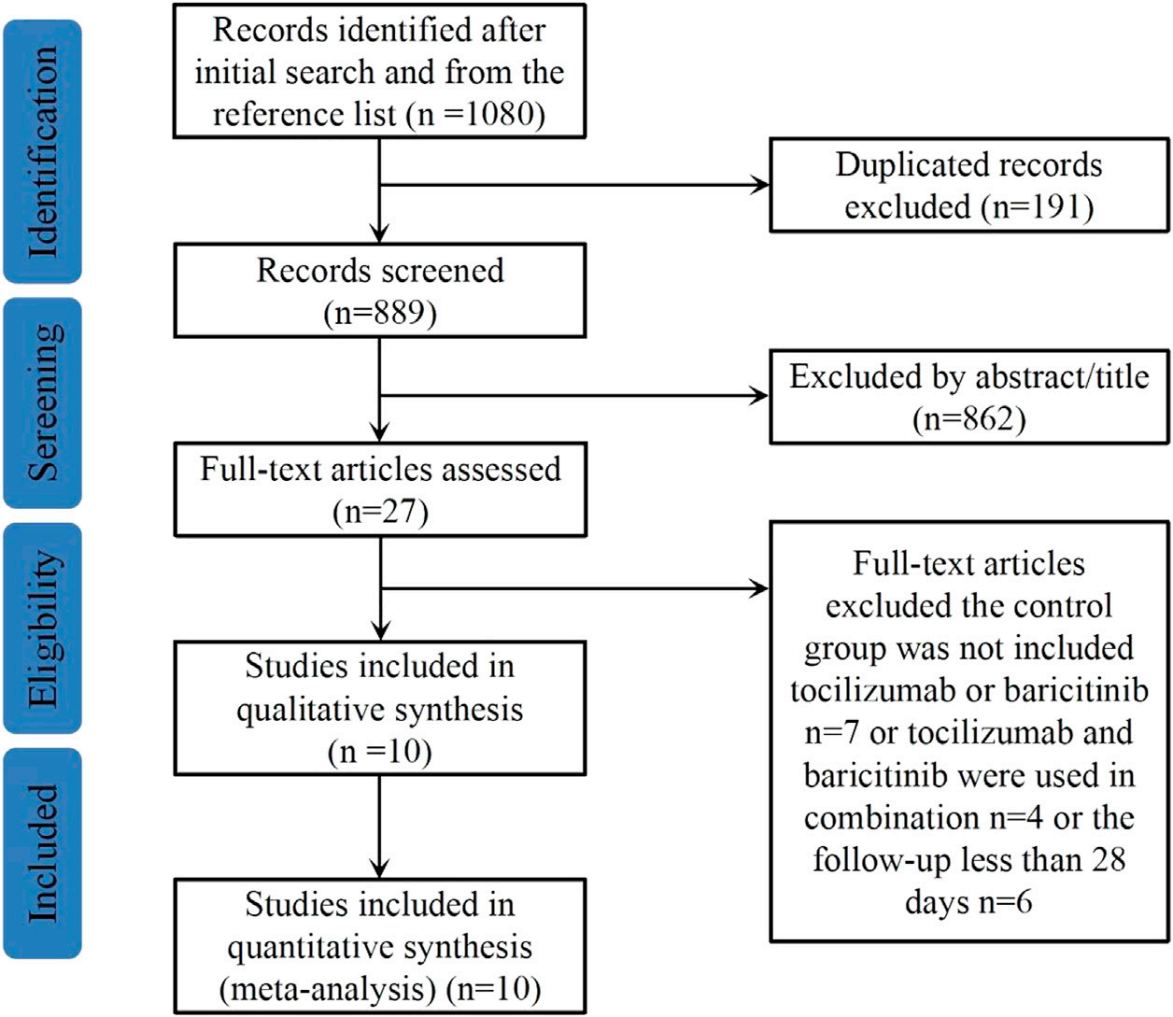
Flow diagram of the literature searching.

The literature search procedure is shown in Figure 1. A total of 1,080 potentially relevant articles were identified from the above databases. After removing 191 duplicated articles, the titles and abstracts of the remaining 889 articles were screened, 862 articles were then excluded as irrelevant and 27 full-text articles were assessed for eligibility. Finally, a total of 10 articles with 2,517 study participants were included in this meta-analysis (Kojima et al., 2022; Lakatos et al., 2022; Reid et al., 2022; Roddy et al., 2022; Rosas et al., 2022; Wong et al., 2022; Karampitsakos et al., 2023; Peterson et al., 2023; Reid et al., 2023; Troyer et al., 2023).

### Study characteristics and quality assessment

**FIGURE 2:**
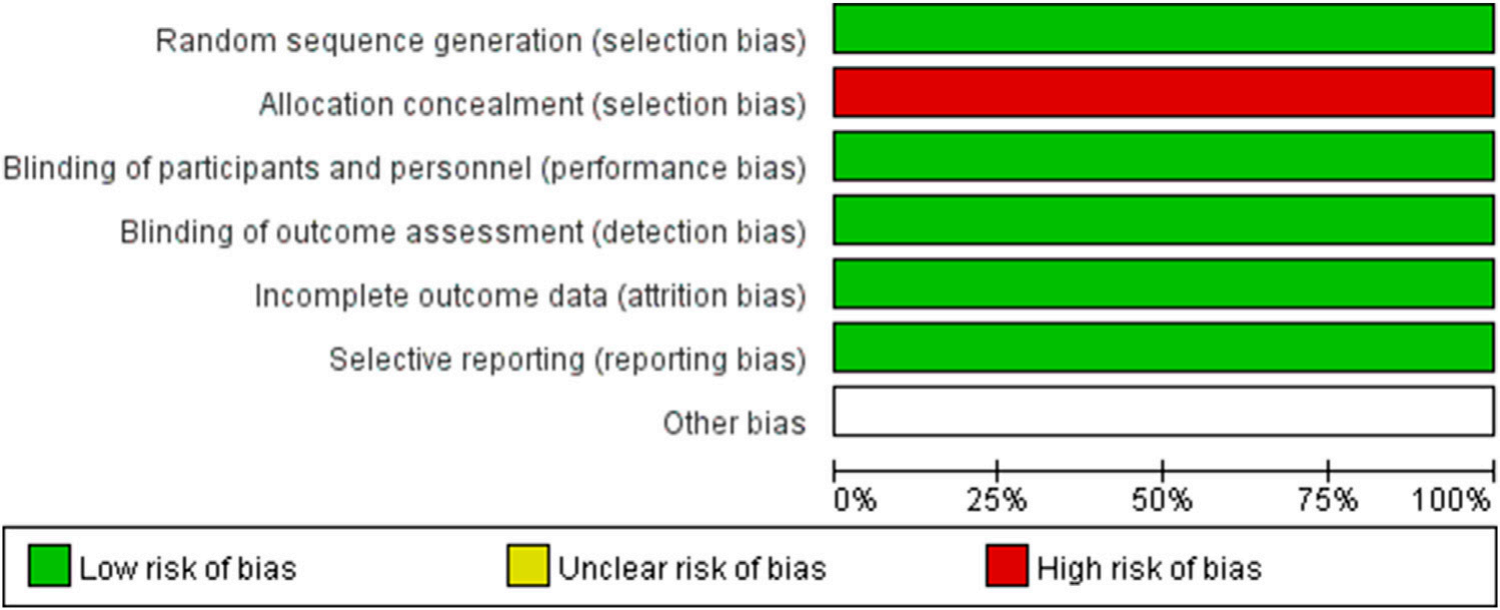
Risk of bias summary in RCT study.

In this meta-analysis with 10 included studies, 1 were RCTs and 9 were cohort studies. The major characteristics of the 10 studies are shown in Table 1. The methodological quality of the RCTs and the comparative cohort studies are shown in Table 2; Figure 2, respectively.

**TABLE 1:** The basic characteristics of eligible studies.

Study	Country	N	Tocilizumab (n)	Baricitinib (n)	Population	Intervention	Outcome	Study design
Lakatos et al. (2022)	Hungary	463	102	361	severe COVID-19	Baricitinib: 4 mg/d orally (7 days min)	in-hospital mortality, ICU length of stay, major infectious complications	cohort study
						Tocilizumab: 8 mg/kg intravenously		
Troyer et al. (2023)	United States	133	64	69	severe COVID-19	Baricitinib: 1–4 mg/d orally, up to 14 days, until discharge or the adverse effects requiring discontinuation	in-hospital mortality, ICU length of stay, major infectious complications	cohort study
						Tocilizumab: 8 mg/kg intravenously, single-dose (max 800 mg)		
Wong et al. (2022)	Hong Kong, China	241	165	76	moderate-to-severe COVID-19	Baricitinib: 4 mg/d orally, up to 14 days or until hospital discharge Tocilizumab: 4–8 mg/kg intravenously, single-dose (max 800 mg for adults)	in-hospital mortality, major infectious complications, severe liver injury, thrombotic and bleeding events	cohort study
Roddy et al. (2022)	United States	382	194	188	COVID-19 Pneumonia and Hypoxemia	Baricitinib: 8 mg/d orally	in-hospital mortality, major infectious complications, thrombotic and bleeding events	cohort study
						Tocilizumab: 800 mg intravenously		
Rosas et al. (2021)	Spain	32	20	12	Pneumonia Secondary to COVID-19	Baricitinib: 2 mg/d or 4 mg/d orally	in-hospital mortality, ICU admission	cohort study
						Tocilizumab: 400 mg in patients weighing <75 kg or 600 mg in those weighing ≥75 kg		
Peterson et al. (2023)	United States	582	291	291	severe COVID-19	Baricitinib: 8 mg/d orally	in-hospital mortality, hospital length of stay, major infectious complications, severe liver injury, thrombotic and bleeding events	cohort study
						Tocilizumab: 8 mg/kg intravenously		
Karolyi et al. (2023)	Austria	159	68	91	severe COVID-19	Baricitinib: 4 mg (GFR>60 mL/min) or 2 mg (GFR 30–60 mL/min) orally for up to 14 days	in-hospital mortality, hospital length of stay, major infectious complications, severe liver injury, thrombotic and bleeding events	cohort study
						Tocilizumab: dose based on body weight (>90 kg		
						800 mg; ≤90 kg: 600 mg; ≤65 kg: 400 mg; ≤40 kg: 8 mg/kg), intravenously		
Reid et al. (2022)	United States	176	61	115	severe COVID-19	Baricitinib: 4 mg/d orally	in-hospital mortality, hospital length of stay, ICU length of stay, major infectious complications, thrombotic and bleeding events	cohort study
						Tocilizumab: 8 mg/kg intravenously, (max 800 mg)		
Karampitsakos et al. (2023)	Greece	251	126	125	severe COVID-19	Baricitinib: 4 mg (GFR>60 mL/min) or 2 mg (GFR 30–60 mL/min) orally for up to 14 days or until hospital discharge	in-hospital mortality, severe liver injury, thrombotic and bleeding events	RCT
						Tocilizumab: 8 mg/kg intravenously		
Kojima et al. (2022)	Japan	98	64	34	moderate-to-severe COVID-19 (89.80%)	Baricitinib: 4 mg (GFR>60 mL/min) or 2 mg (GFR 30–60 mL/min) orally for up to 14 days or the patient’s symptoms had improved	in-hospital mortality, major infectious complications	cohort study
						Tocilizumab: 8 mg/kg intravenously		

**TABLE 2:** Quality assessment of eligible studies.

Cochrane Collaboration’s tool	Random sequence generation	Allocation concealment	Blinding of participants and personnel	Blinding of outcome assessment	Incomplete outcome data	Selective reporting	Other sources of bias						
RCT													
Kojima et al. (2022)	Low risk	High risk	Low risk	Low risk	Low risk	Low risk	Unclear risk						

MINORS	A clearly stated aim	Inclusion of consecutive patients	Prospective collection of data	Endpoints appropriate to the aim of the study	Unbiased assessment of the study endpoint	Follow-up period appropriate to the aim of the study	Loss to follow up less than 5%	Prospective calculation of the study size	An adequate control group	Contemporary groups	Baseline equivalence of groups	Adequate statistical analyses	Score
Lakatos et al. (2022)	2	2	2	2	2	2	2	0	2	2	2	2	22
Troyer et al. (2023)	2	1	0	2	0	1	1	0	2	2	2	1	14
Wong et al. (2022)	2	2	0	2	2	2	2	0	2	2	2	2	20
Roddy et al. (2022)	2	1	0	2	0	2	2	0	2	2	1	1	15
Rosas et al. (2022)	2	2	0	2	1	2	2	0	1	1	1	1	15
Peterson et al. (2023)	2	2	0	2	1	2	2	0	2	2	2	2	19
Karolyi et al. (2023)	2	2	0	2	0	1	2	0	2	2	1	1	15
Reid et al. (2022)	2	2	0	2	0	2	2	0	1	2	1	1	15
Kojima et al. (2022)	2	2	0	2	2	2	2	0	1	1	1	2	17

### Mortality rate

**FIGURE 3:**
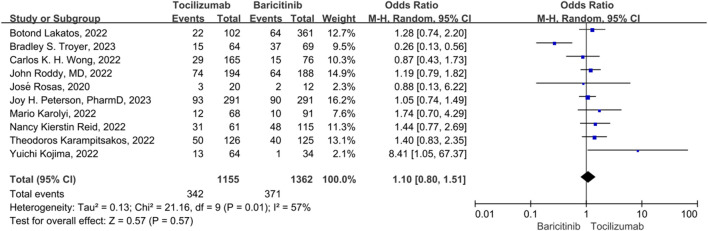
The mortality rate between tocilizumab and baricitinib in patients with COVID-19.

For mortality, 10 studies were included. The heterogeneity between the 10 studies was statistically different (*p* = 0.01, *I* ^*2*^ = 57%), so a random-effect model was used. There was no statistically significant difference in the 28 days mortality rate between the tocilizumab and baricitinib (OR = 1.10, 95% CI = 0.80–1.51, *p* = 0.57) (Figure 3).

### Hospital length of stay

**FIGURE 4:**
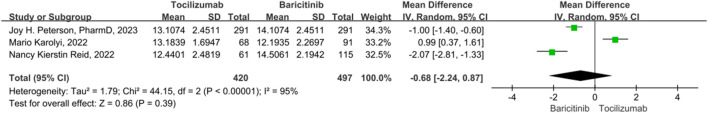
The hospital length of stay between tocilizumab and baricitinib in patients with COVID-19.

For length of hospital stay, 3 studies were included. A random model was employed, in that significant heterogeneity was observed among the study (*p* < 0.01, *I* ^*2*^ = 95%). For hospital length of stay, there was no statistically significant difference between the tocilizumab and baricitinib (OR = −0.68, 95% CI = −2.24–0.87, *p* = 0.39) (Figure 4).

### ICU length of stay

**FIGURE 5:**
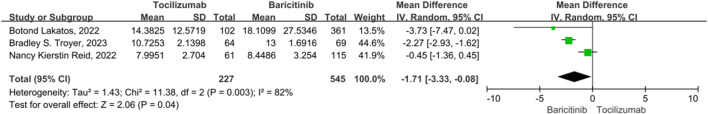
The ICU length of stay between tocilizumab and baricitinib in patients with COVID-19.

There were 3 studies on the ICU length of stay, with significant heterogeneity (*p* = 0.03, *I* ^*2*^ = 82%). The combined results indicate that tocilizumab is associated with a statistically significant reduction in ICU length of stay compared to baricitinib. (OR = −1.71, 95% CI = −3.33–−0.08, *p* = 0.04) (Figure 5).

### Secondary infection rate

**FIGURE 6:**
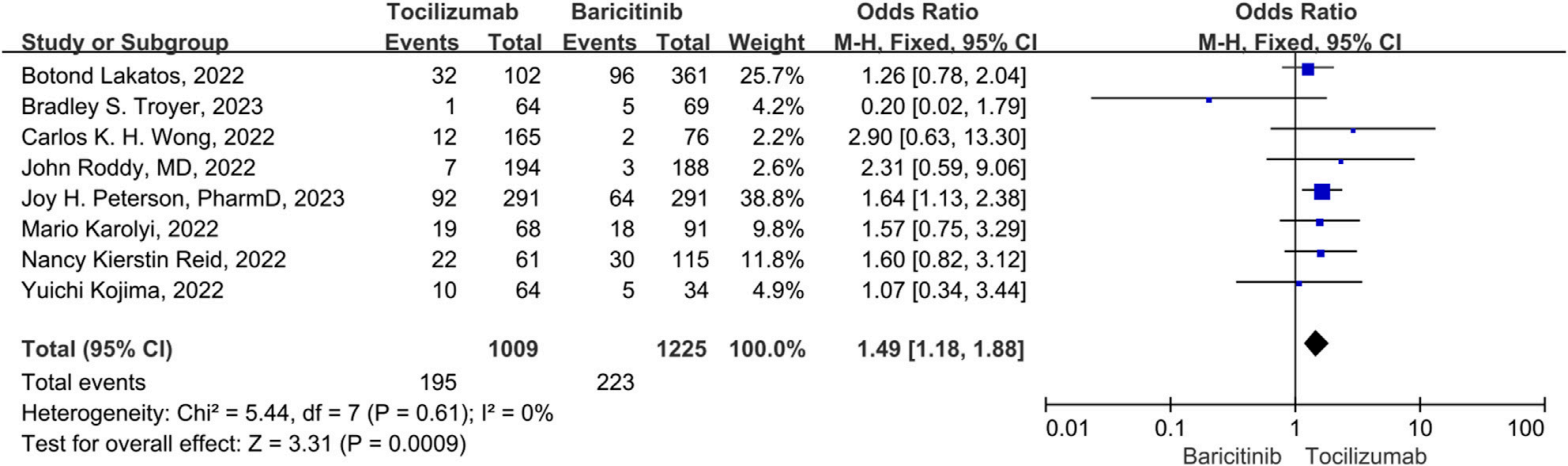
The secondary infection rate between tocilizumab and baricitinib in patients with COVID-19.

Five studies were included in the analysis of secondary infection rates, and no significant heterogeneity was observed among them (*p* = 0.61, I^2^ = 0%). Therefore, a fixed-effect model was used to combine the results. The results indicated that tocilizumab had a significantly higher secondary infection rate compared to baricitinib (OR = 1.49, 95% CI = 1.18–1.88, *p* < 0.001) (Figure 6).

### Thrombotic and bleeding

**FIGURE 7:**
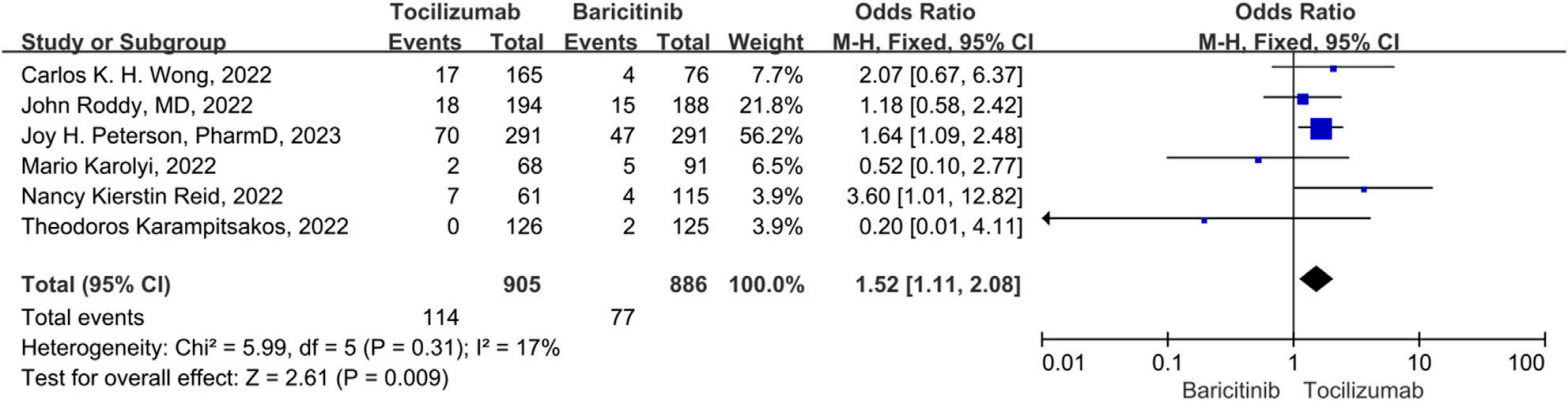
Thrombotic and bleeding events between tocilizumab and baricitinib in patients with COVID-19.

There were 3 studies included in the thrombotic and bleeding events. No significant heterogeneity was observed among the study (*p* = 0.31, *I* ^*2*^ = 17%), thus a fix-effect model was used to pool the outcomes for studies. The results showed that the thrombotic and bleeding events of tocilizumab significantly was higher than that of the baricitinib (OR = 1.52, 95% CI = 1.11–2.08, *p* = 0.009) (Figure 7).

### Acute liver injury

**FIGURE 8:**
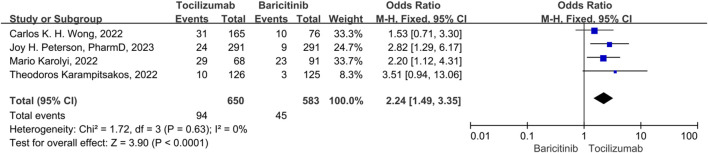
Acute liver injury between tocilizumab and baricitinib in patients with COVID-19.

There were 4 studies included in the acute liver injury events. A fix-effect model was employed, in that no significant heterogeneity was observed among the studies (*p* = 0.31, *I* ^*2*^ = 17%). The results indicated that the acute liver injury events of tocilizumab was significantly higher than that of the baricitinib (OR = 2.24, 95% CI = 1.49–3.35, *p* < 0.001) (Figure 8).

### Sensitivity analysis

**FIGURE 9:**
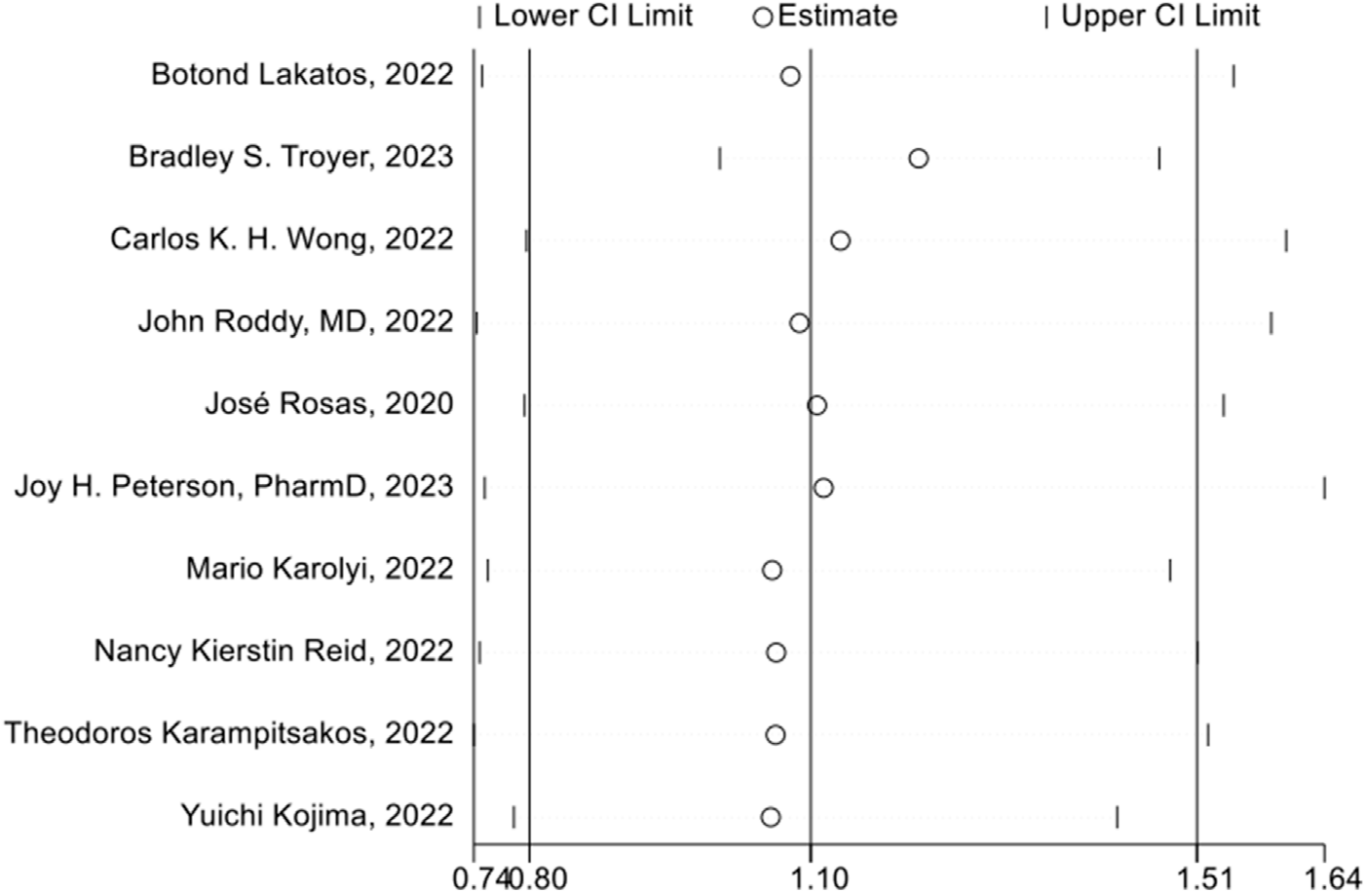
Sensitivity analysis of 28 days mortality rate between tocilizumab and baricitinib in patients with COVID-19.

The sensitivity analysis was assessed using a one-by-one elimination method, and the overall findings of the remaining research were statistically unchanged after methodically removing each piece of literature. This indicated that the results of our study were stable (Figure 9).

### Publication bias

**FIGURE 10:**
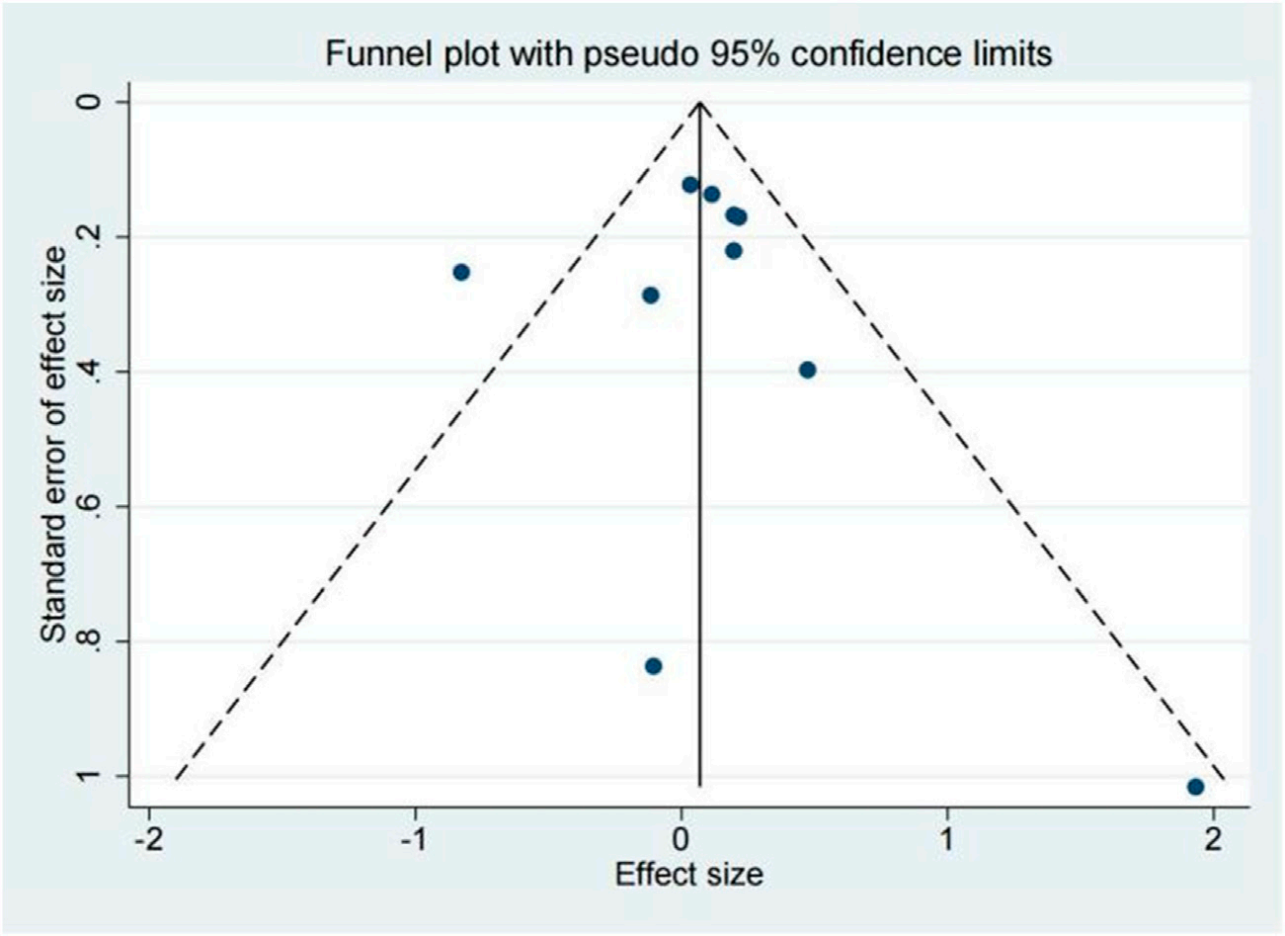
Funnel plot of 28 days mortality rate between tocilizumab and baricitinib in patients with COVID-19.

**FIGURE 11:**
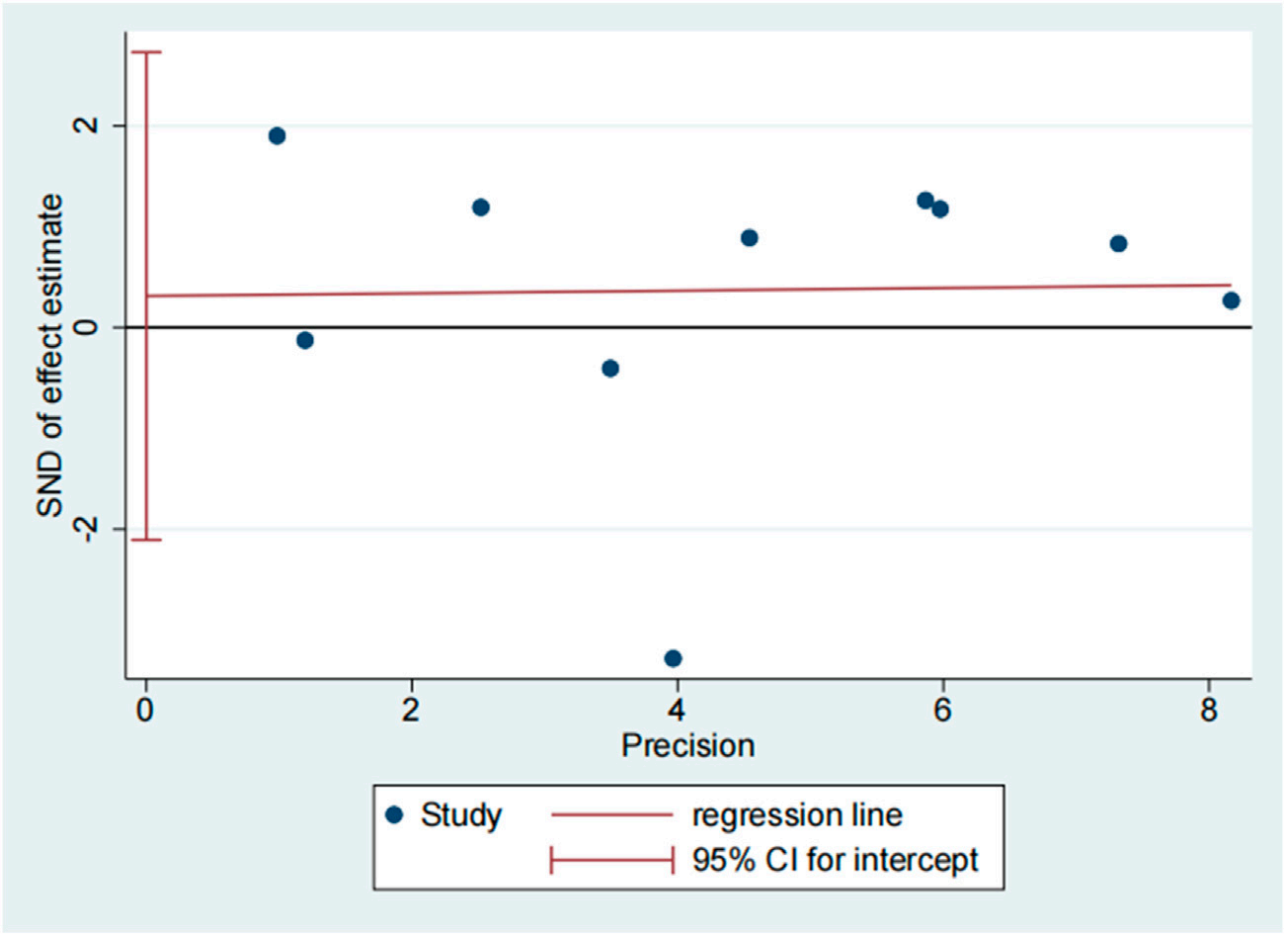
Egger’s test of 28 days mortality rate between tocilizumab and baricitinib in patients with COVID-19.

The publication bias of the included studies was evaluated by using funnel plots and Egger’s tests. As no asymmetry of the funnel plot was observed (Figure 10), the plots and the Egger’s test suggested that there was absence of publication in this meta-analysis (t = 0.30, 95% CI = −2.11 to 2.73, *p* = 0.773) (Figure 11; Table.3).

**TABLE 3:** Egger’s test of 28 days mortality rate between tocilizumab and baricitinib in patients with COVID-19.

Std_Eff	Coefficient	Std.Err	T	*p* > |t |	95%CI
Slope	0.0132266	0.2107956	0.06	0.952	−0.4728691, 0.4993222
Bias	0.312877	1.049108	0.30	0.773	−2.10637, 2.732124

## Discussion

### Summary of results

Although the WHO’s determination that COVID-19 no longer constitutes a PHEIC, it remains an ongoing health issue with a monthly death toll approaching 20,000 worldwide (World Health Organization, 2005). Systemic inflammatory response syndrome is a significant contributor to mortality, and immunomodulatory drugs such as tocilizumab and baricitinib have been widely used in conjunction with dexamethasone (World Health Organization, 2021). Both tocilizumab and baricitinib are thought to be beneficial and are recommended in this patient population, but the relative merits of each have not previously been evaluated. To the best of our knowledge, this is the first meta-analysis to directly evaluate the efficacy and safety of tocilizumab and baricitinib in COVID-19 patients with hyperinflammatory response. In order to compare the therapeutic efficacy of tocilizumab and baricitinib in COVID-19 patients with a hyperinflammatory response, we employed 28 days mortality rate, hospital length of stay, and ICU length of stay as evaluation index. The results of this study showed that there was no statistical difference in 28-day mortality rate (29.61% vs. 27.24%, *p* = 0.57) and hospital length of stay (*p* = 0.39) between tocilizumab and baricitinib. However, tocilizumab may result in a shorter ICU stay compared to baricitinib (*p* = 0.04), possibly due to the slightly higher mortality rate associated with tocilizumab.

Otherwise, we conducted a comparative analysis of the safety profiles of tocilizumab and baricitinib in the treatment of COVID-19 patients in terms of secondary infections, thrombotic and hemorrhagic events, as well as acute liver injury. The results show that the secondary infection rate in tocilizumab were higher than baricitinib among COVID-19 patients (*p* < 0.001). In addition, compared to baricitinib, tocilizumab may cause greater thrombotic and bleeding events (*p* = 0.009). Furthermore, tocilizumab caused acute liver damage more frequently than baricitinib (*p* < 0.001).

### Studies from the literature

The previous systematic review demonstrated that both tocilizumab and baricitinib, when compared to standard of care, can reduce the mortality rates and hospital length of stay in patients with predominantly moderate-to-severe COVID-19 (Alkofide et al., 2021; Tharmarajah et al., 2021; Walz et al., 2021; WHO Rapid E et al., 2021). However, there is a lack of head-to-head studies comparing the efficacy and safety of these two treatments. Based on its superior 28-day mortality data, ease of administration, shorter half-life, and lower cost of treatment, baricitinib may be preferred over tocilizumab (Cherian et al., 2022). This finding is consistent with a network meta-analysis that showed no statistically significant difference in mortality rate between the two drugs (Ngamprasertchai et al., 2022).

The JAK-STAT signaling pathway is central to the development of the cytokine storm in COVID-19, which could activate cytokine levels including IL6, IL2, interferon-gamma, etc., (Limen et al., 2022). Baricitinib modulates downstream inflammatory responses via JAK1/JAK2 inhibition and IL-6-induced STAT3 phosphorylation. The anti-cytokine and anti-viral activities of baricitinib are responsible for a rapid reduction in the viral load, inflammatory markers, and IL-6 levels in COVID-19 (Zhang et al., 2020). IL-6 is a pleiotropic cytokine secreted by neutrophils, monocytes, and macrophages and involved in the inflammatory response. This in turn causes impaired oxygen diffusion, and the ensuing inflammation eventually leads to lung fibrosis and multi-organ failure (Gordon et al., 2021). Furthermore, elevated levels of IL-6 have been associated with a hypercoagulable state in patients with COVID-19. Tocilizumab competitively binds to the IL-6 receptor, thereby blocking IL-6-mediated signaling and preventing the assembly of the activated complex with transmembrane proteins. This mitigates immune-mediated damage, lung injury, and reduces oxygen saturation. (Kalil et al., 2021; Rosas et al., 2021; Tharmarajah et al., 2021).

The safety between tocilizumab and baricitinib were still controversial. Numerous prior studies have indicated a lack of substantial disparity in terms of safety between the aforementioned groups; nevertheless, certain recent studies have presented contrasting perspectives. A network meta-analysis was conducted to assess the efficacy and safety of immunomodulators in patients with COVID-19, revealing that tocilizumab may pose a higher risk of infection compared to baricitinib (Ngamprasertchai et al., 2022). Other studies have additionally indicated an elevation in the incidence of thrombosis or acute liver injury among individuals administered tocilizumab as opposed to baricitinib (Karolyi et al., 2023; Peterson et al., 2023; Reid et al., 2023). Our study found that the tocilizumab group had adverse effects more frequently, including secondary infections, thrombotic events, and acute liver damage. The lower rate of adverse effects seen in our study provides valuable insight into the relative safety of short-term use of baricitinib for severe COVID-19. Notably, the pharmacologic half-life of baricitinib is 12 h and that of tocilizumab is up to 13 days (U.S. Food and Drug Administration, 2018; U.S. Food and Drug Administration, 2022)Tocilizumab exhibits a notable capacity to effectively attenuate fever and immune responses triggered by infection for a duration of one or 2 week. Consequently, it is plausible that the incidence of early superinfection might be underestimated (Peterson et al., 2023). Furthermore a single intravenous dose may result in a higher peak blood concentration compared to a daily oral dose, thereby increasing the risk of adverse effects. In addition, although tocilizumab is recommended as a one-time IV infusion for this indication, the full recommended course of baricitinib is 14 days. The duration of baricitinib treatment occasionally fell below a fortnight Thus, a more prolonged drug effect may explain the increased adverse effects seen with tocilizumab.

### Strengths and limitations

Our study had several strengths. First, we performed a direct comparison between tocilizumab and baricitinib, which is more compelling than previous studies. Second, our methodology was strictly adhered to the Cochrane Handbook, and the protocol was registered in PROSPERO. Lastly, the number of included articles was the largest, and the evaluation indicators covered efficacy and safety. However, some limitations of our study must be addressed. First, the results were heterogeneous across the included studies. However, Some included studies had inconsistent follow-up time and varying criteria for evaluating efficacy and safety.

In addition, although we did not explore the dominant COVID-19 variants at the time of each study, the effects of variants on the treatment response to immunomodulators were inconclusive. Third, countries or regions might be confounding factors. However, as included studies were multinational and mixed populations were recruited, these will minimize the threat to the validity of this study. Finally, heterogeneity concerning the dosage, timing, and duration of immunomodulator therapy should be further explored, particularly in large-scale clinical trials.

## Conclusion

We conducted a meta-analysis to evaluate the efficacy and safety among tocilizumab and baricitinib in treating severe COVID-19 patients. The results showed no difference in the in-hospital mortality rate or hospital length of stay between the two treatments. However, the baricitinib group experienced significantly fewer adverse effects. Although further prospective, randomized trials are needed to further assess this association, our data suggest that baricitinib may be a better choice for treating severe COVID-19 patients.

## Data Availability

The original contributions presented in the study are included in the article/Supplementary Materials, further inquiries can be directed to the corresponding author/s.
