# Supplementary material for: Efficacy and safety of tocilizumab and baricitinib among patients hospitalized for COVID-19: a systematic review and meta-analysis
Source: Front Pharmacol. 2023 Nov 22;14:1293331. doi: 10.3389/fphar.2023.1293331 (PMC10703388; doi:10.3389/fphar.2023.1293331)
Supplement: Supplementary file 1 [file DataSheet1.docx]

Supplementary Material

**Supplementary Table 1. Literature search strategy.**

| Patients | COVID-19 | COVID-19 OR (COVID-19 Virus Disease) OR (COVID-19 Virus Disease) OR (COVID-19 Virus) OR (Virus Disease, COVID-19) OR (COVID-19 Virus Infection) OR (Infection, COVID-19 Virus) OR (2019-nCoV Infection) OR (2019 nCoV Infection) OR (2019-nCoV Infections) OR (Infection, 2019-nCoV) OR (Coronavirus Disease-19) OR (Coronavirus Disease 2019) OR (2019 Novel Coronavirus Disease) OR (2019 Novel Coronavirus Infection) OR (2019-nCoV Disease) OR (2019 nCoV Disease) OR (2019-nCoV Diseases) OR (Disease, 2019-nCoV OR COVID19) OR (Coronavirus Disease 2019) OR (Disease 2019, Coronavirus) OR (SARS Coronavirus 2 Infection) OR (SARS-CoV-2 Infection) OR (Infection, SARS-CoV-2) OR (SARS CoV 2 Infection) OR (SARS-CoV-2 Infections) OR (COVID-19 Pandemic) OR (COVID-19 Pandemic) OR (COVID-19 Pandemics) OR (Pandemic, COVID-19) |
| --- | --- | --- |
| Intervention | Tocilizumab | tocilizumab OR Actemra |
|  | Baricitinib | baricitinib OR Olumiant |
